# Crystal structures of 4-methyl-2-oxo-2*H*-chromene-7,8-diyl di­acetate and 4-methyl-2-oxo-2*H*-chromene-7,8-diyl bis­(pent-4-ynoate)

**DOI:** 10.1107/S2056989016005892

**Published:** 2016-04-15

**Authors:** Akintunde Akinyemi, Courtney Thomas, Willis Marsh, Ray J. Butcher, Jerry P. Jasinski, Lystranne A. Maynard-Smith

**Affiliations:** a Department of Chemistry, Howard University, 525 College Street NW, Washington, DC 20059, USA; b Department of Chemistry, Keene State College, 229 Main Street, Keene, NH 03435-2001, USA

**Keywords:** crystal structure, coumarin, pent-4-ynoate substituent, acetate substituent

## Abstract

In the structures of the two title coumarin derivatives, C_14_H_12_O_6_, (**1**), and C_20_H_16_O_6_, (**2**), one with acetate and the other with pent-4-ynoate substituents, both the coumarin rings are almost planar. In (**1**), both acetate substituents are significantly rotated out of the coumarin plane to minimize steric repulsions. One acetate substituent is disordered over two equivalent conformations, with occupancies of 0.755 (17) and 0.245 (17). In (**2**), there are two pent-4-ynoate substituents, the C≡C group of one being disordered over two positions with occupancies of 0.55 (2) and 0.45 (2). One of the pent-4-ynoate substituents is in an extended conformation, while the other is in a bent conformation. In this derivative, the planar part of both pent-4-ynoate substituents deviate from the coumarin plane. The packing of (**1**) is dominated by π–π stacking involving the coumarin rings and weak C—H⋯O contacts link the parallel stacks in the [101] direction. In contrast, in (**2**) the packing is dominated by *R*_2_^2^(24) hydrogen bonds, involving the acidic *sp* H atom and the oxo O atom, which link the mol­ecules into centrosymmetric dimers. The bent conformation of one of the pent-4-ynoate substituents prevents the coumarin rings from engaging in π–π stacking.

## Abstract 2

The structures of two substituted coumarin derivatives are reported, one with acetate and the other with pent-4-ynoate substituents.

## Chemical context   

Coumarins and their derivatives have wide applications in a number of diverse areas. They are used in the pharmaceutical industry as precursor reagents in the synthesis of a number of synthetic anti­coagulant pharmaceuticals (Bairagi *et al.*, 2012 ▸), the most notable being warfarin (Holbrook *et al.*, 2005 ▸). Modified coumarins are a type of vitamin K antagonist (Marongiu & Barcellona, 2015 ▸).

In another important application, coumarin dyes are extensively used as gain media in blue–green tunable organic dye lasers (Schäfer, 1990 ▸; Duarte & Hillman, 1990 ▸; Duarte, 2003 ▸). Coumarin tetra­methyl laser dyes offer wide tunability and high laser gain (Chen *et al.*, 1988 ▸; Duarte *et al.*, 2006 ▸), and they are also used as the active medium in coherent OLED emitters (Duarte *et al.*, 2005 ▸).

4-Methyl coumarin derivatives have previously been used as acetyl-group donors for post-translational modification of proteins *via* an acet­yl–CoA independent mechanism (Raj, Singh *et al.*, 2005 ▸; Raj, Kumari *et al.*, 2006 ▸). Calreticulin-mediated acetyl­ation of gluta­thione-S-transferase (GST) using substrate 7,8-diacety­oxy-4-methyl coumarin, DAMC (**1**) (systematic name: 4-methyl-2-oxo-2*H*-chromene-7,8-diyl di­acetate) has been shown to inhibit GST activity in a spectroscopic assay (Raj, Singh *et al.*, 2005 ▸). The crystal structure of the related compound 7,8-dihy­droxy-4-methyl­coumarin (Kurosaki *et al.*, 2003 ▸) has been reported. Pentynoyl probes have been used as chemical reporters to monitor protein acetyl­ation (Bateman *et al.*, 2013 ▸; Yang *et al.*, 2010 ▸). For background to bio-orthogonal reactions using alkyne–azide cyclo­addition, see Sletten & Bertozzi (2011 ▸) and Yang & Hang (2011 ▸).
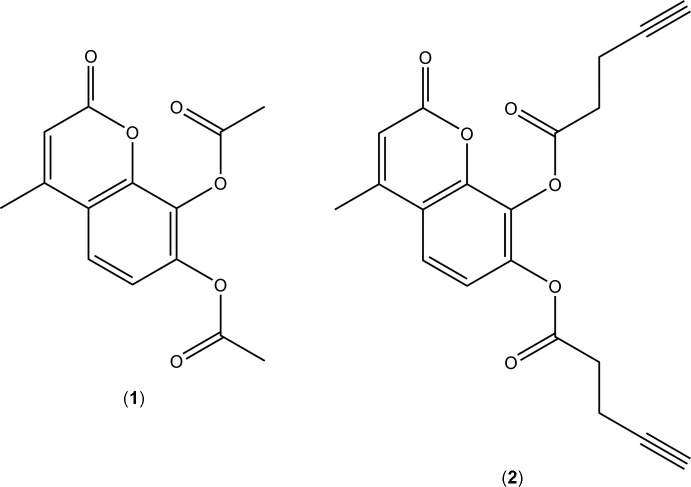


We have synthesized a new coumarin derivative, 7,8-dipentyno­yloxy-4-methyl coumarin, DPeMC (**2**) [systematic name: 4-methyl-2-oxo-2*H* chromene-7,8-diyl bis­(pent-4-ynoate)] as a chemical reporter of calreticulin’s acyl­transferase capabilities (Singh *et al.*, 2011 ▸). As part of this work, the crystal structures of both coumarin derivatives are presented in this article.

## Structural commentary   

This paper reports the structures of two derivatives of coumarin (systematic name; 2*H*-chromen-2-one), C_14_H_12_O_6_ (**1**) and C_20_H_16_O_6_ (**2**), which are to be used as chemical reporters of calreticulin’s acyl­transferase capabilities. These two compounds will be first discussed individually and then compared.

**Figure 1:**
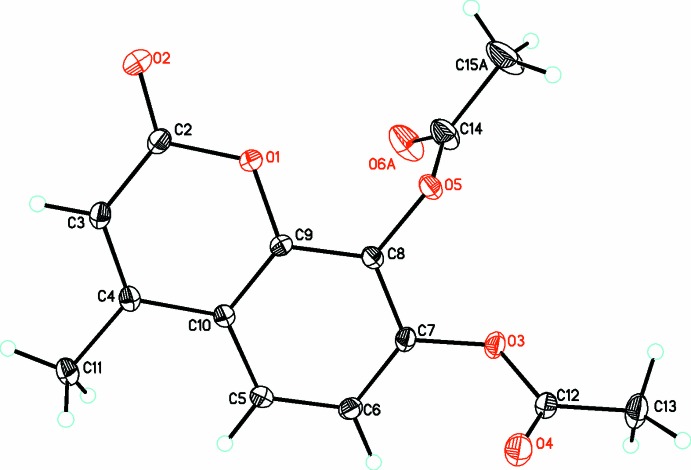
Diagram of the structure and numbering scheme for (**1**), showing the major occupancy component only. Atomic displacement parameters are drawn at the 30% probability level.

In the structure of (**1**) (Fig. 1 ▸), the coumarin ring is almost planar (r.m.s. deviation of fitted atoms = 0.0063 Å) with O2 in the plane [deviation of 0.0048 (9) Å]. Both acetate substituents are significantly rotated out of this plane to minimize steric repulsions [dihedral angle of 66.19 (7)° to the coumarin ring for O3, O4, and C11, and 79.4 (3)° for O5, C13 O6*A*]. One acetate substituent is disordered over two equivalent conformations with occupancies of 0.755 (17) and 0.245 (17). The metrical parameters of both the coumarin ring and acetate substituents are in the normal ranges.

**Figure 2:**
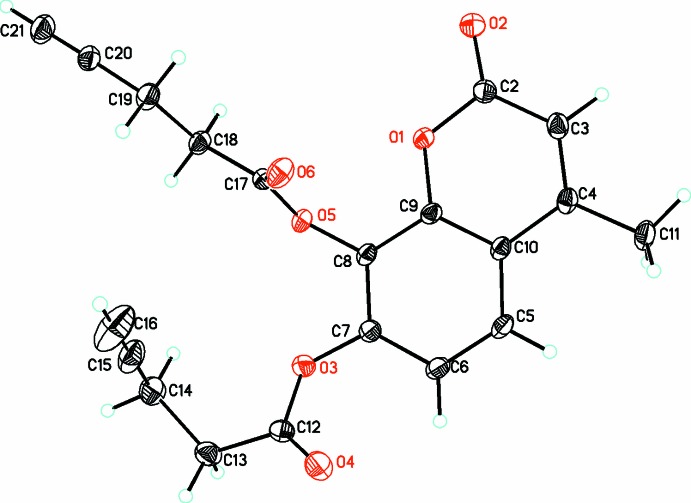
Diagram of the structure and numbering scheme for (**2**), showing the major occupancy component only. Atomic displacement parameters are drawn at the 30% probability level.

In (**2**) (Fig. 2 ▸), the C≡C group of one of the pent-4-ynoate substituents is disordered over two positions with occupancies of 0.55 (2) and 0.45 (2). The coumarin ring is almost planar (r.m.s. deviation of fitted atoms = 0.0305 Å) with O2 significantly out of this plane [0.144 (2) Å] but O3 in the plane [0.063 (2) Å]. One of the pent-4-ynoate substituents is in an extended conformation (O5 to C21) while the other is in a bent conformation about C13. This can be seen from a consideration of the O3—C12—C13—C14 torsion angle of −46.3 (2)° compared to the equivalent torsion angle O5—C17—C18—C19 of 176.16 (12)°. The planar parts of both pent-4-ynoate substituents deviate from the coumarin plane but by different amounts [40.90 (15)° for O3, O4 and C12 compared to 74.07 (10)° for O5, O6 and C17]. The metrical parameters of both the coumarin ring and pent-4-ynoate substituents are in the normal ranges including the C≡C triple bonds [C15*A*≡C16*A* = 1.186 (9), C15*B*≡C16*B* = 1.169 (11) and C20≡C21 = 1.177 (3) Å].

## Supra­molecular features   

**Figure 3:**
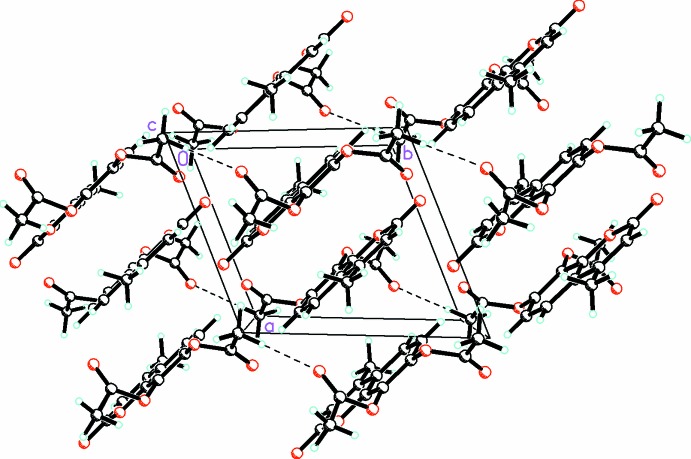
Packing diagram for (**1**), viewed along the *c* axis, showing the parallel coumarin rings. C—H⋯O secondary inter­actions are drawn with dashed lines.

The packing of (**1**) is dominated by π–π stacking involving the coumarin rings [centroid–centroid distance of 3.6640 (5) Å, slippage of 1.422 Å, symmetry code 1 − *x*, 1 − *y*, 1 − *z*]. This can be observed in Fig. 3 ▸. In addition, there are weak C—H⋯O contacts (Table 1 ▸) involving C13 and O6*A*(*x*, 1 + *y*, *z*) as well as C6 and O2(*x* − 1, 1 + *y*, *z*), C15*A* and O2 (1 − *x*, −*y*, 2 − *z*) which link the parallel stacks in the [101] direction.

**Figure 4:**
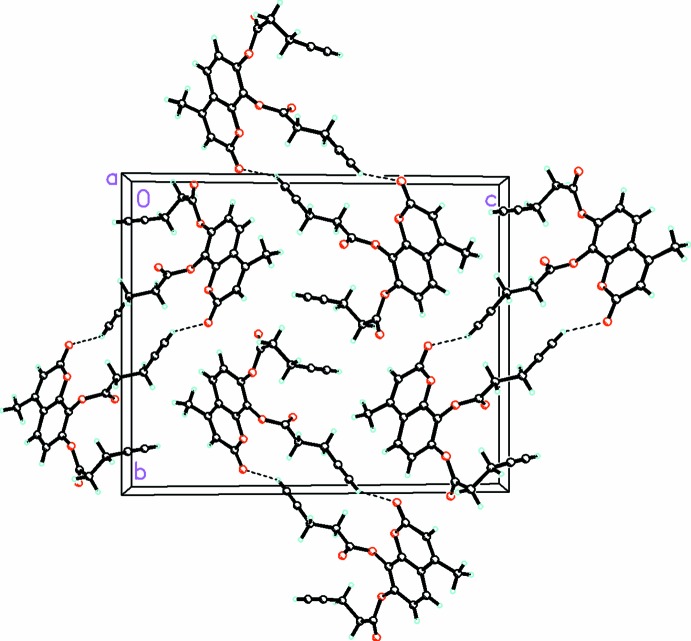
Packing diagram for (**2**), viewed along the *a* axis. 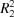
(24) hydrogen bonds involving the acidic *sp* H and O2 atoms link the mol­ecules into centrosymmetric dimers. C—H⋯O secondary inter­actions are drawn with dashed lines.

In contrast to (**1**), for (**2**) the packing (Fig. 4 ▸) is dominated by 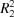
(24) hydrogen bonds (Table 2 ▸) involving the acidic *sp* H atom and O2 which link the mol­ecules into centrosymmetric dimers. The bent conformation of one of the pent-4-ynoate substituents prevents the coumarin rings from engaging in π–π stacking in contrast to (**1**).

## Database survey   

Our group has reported a number of related structures (Jasinski & Paight, 1994 ▸, 1995 ▸; Jasinski & Woudenberg, 1994 ▸, 1995 ▸; Jasinski & Li, 2002 ▸; Jasinski *et al.*, 1998 ▸, 2003 ▸; Butcher *et al.*, 2007 ▸).

## Synthesis and crystallization   

*7,8-Diacet­oxy-4-methyl­coumarin* (**1**). 4-Methyl-2-oxo-2*H*-chromene-7,8-diyl di­acetate (DAMC) was synthesized using a previously reported procedure (Jalal *et al.*, 2012 ▸).

*7,8-Dipentyno­yloxy-4-methyl­coumarin* (**2**). 0.5 mmol 7,8-dihy­droxy-4-methyl coumarin, DHMC [systematic name: 7,8-dihy­droxy-4-methyl-2*H*-chromen-2-one], 2.5 equivalents pentynoic anhydride (Malkoch *et al.*, 2005 ▸) and catalytic 4-di­methyl­amino­pyridine (DMAP) was stirred for 24 h at room temperature in anhydrous THF (2 mL). Ice-cold water (25 mL) was added to the reaction flask, and the filtered crude product was washed with hexa­nes followed by recrystallization from ethanol to obtain small brown crystals of 4-methyl-2-oxo-2*H* chromene-7,8-diyl bis­(pent-4-ynoate).

Spectroscopic analysis: ^1^H NMR (400 MHz, CDCl_3_): δ 7.51–7.49 (1H, *d*), δ 7.20–7.17 (1H, *d*), δ 6.29 (1H, *s*), δ 3.01–3.08 (2H, *m*, HC≡C), δ 2.89–2.84 (2H, *t*, C≡C—CH_2_), δ 2.61–2.70 (4H, *m*, OOC—CH_2_), δ 2.44 (3H, *s*, CH_3_), δ 2.09–2.11 (2H, C≡C-*–*CH_2_).

## Refinement   

Crystal data, data collection and structure refinement details are summarized in Table 3 ▸. For (**1**), the H atoms were positioned geometrically and refined as riding: C—H = 0.95–0.98 Å with *U_iso_*(H) = 1.5*U*_eq_(C) for methyl H atoms and = 1.2*U_eq_*(C) for other H atoms. One acetate substituent is disordered over two equivalent conformations with occupancies of 0.755 (17) and 0.245 (17).

In the refinement for (**2**), the H atoms were positioned geometrically and refined as riding: C—H = 0.95–0.99 Å with *U*_iso_(H) = 1.5*U*_eq_(C) for methyl H atoms and = 1.2*U_eq_*(C) for other H atoms. The C≡C group of one of the pent-4-ynoate substituents is disordered over two positions with occupancies of 0.55 (2) and 0.45 (2).

## Supplementary Material

Crystal structure: contains datablock(s) 1, 2. DOI: 10.1107/S2056989016005892/hg5471sup1.cif

Structure factors: contains datablock(s) 1. DOI: 10.1107/S2056989016005892/hg54711sup2.hkl

Structure factors: contains datablock(s) 2. DOI: 10.1107/S2056989016005892/hg54712sup3.hkl

Supporting information file. DOI: 10.1107/S2056989016005892/hg54711sup4.cml

CCDC references: 1473151, 1473150

Additional supporting information:  crystallographic information; 3D view; checkCIF report

## Figures and Tables

**Table 1:** Hydrogen-bond geometry (Å, °) for (**1**)

*D*—H⋯*A*	*D*—H	H⋯*A*	*D*⋯*A*	*D*—H⋯*A*
C6—H6*A*⋯O2^i^	0.95	2.65	3.3465 (17)	130
C13—H13*A*⋯O6*A* ^ii^	0.98	2.48	3.451 (5)	173
C15*A*—H15*B*⋯O2^iii^	0.98	2.52	3.401 (8)	150

Symmetry codes: (i) 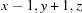; (ii) 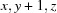; (iii) 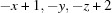.

**Table 2:** Hydrogen-bond geometry (Å, °) for (**2**)

*D*—H⋯*A*	*D*—H	H⋯*A*	*D*⋯*A*	*D*—H⋯*A*
C13—H13*B*⋯O4^i^	0.99	2.43	3.244 (2)	139
C18—H18*A*⋯O6^i^	0.99	2.51	3.482 (2)	167

Symmetry code: (i) 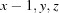.

**Table 3:** Experimental details

	(**1**)	(**2**)
Crystal data		
Chemical formula	C_14_H_12_O_6_	C_20_H_16_O_6_
*M*_r_	276.24	352.33
Crystal system, space group	Triclinic, *P* 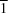	Monoclinic, *P*2_1_/*n*
Temperature (K)	173	200
*a*, *b*, *c* (Å)	7.3722 (10), 8.7235 (7), 11.7032 (15)	5.2785 (3), 16.3785 (8), 20.0502 (11)
α, β, γ (°)	69.263 (10), 87.519 (11), 69.113 (10)	90, 95.992 (2), 90
*V* (Å^3^)	654.66 (14)	1723.95 (16)
*Z*	2	4
Radiation type	Mo *K*α	Mo *K*α
μ (mm^−1^)	0.11	0.10
Crystal size (mm)	0.33 × 0.26 × 0.11	0.55 × 0.14 × 0.11

Data collection		
Diffractometer	Agilent Xcalibur Eos Gemini	Bruker Quest
Absorption correction	Multi-scan (*CrysAlis PRO*; Agilent, 2014 ▸)	Multi-scan (*SADABS*; Sheldrick, 1996 ▸)
*T*_min_, *T*_max_	0.883, 1.000	0.658, 0.746
No. of measured, independent and observed [*I* > 2σ(*I*)] reflections	7360, 4296, 3087	24358, 5276, 3859
*R*_int_	0.036	0.035
(sin θ/λ)_max_ (Å^−1^)	0.759	0.716

Refinement		
*R*[*F* ^2^ > 2σ(*F* ^2^)], *wR*(*F* ^2^), *S*	0.055, 0.156, 1.04	0.057, 0.142, 1.07
No. of reflections	4296	5276
No. of parameters	192	255
No. of restraints	13	13
H-atom treatment	H-atom parameters constrained	H-atom parameters constrained
Δρ_max_, Δρ_min_ (e Å^−3^)	0.36, −0.24	0.37, −0.21

Computer programs: *CrysAlis PRO* (Agilent, 2014 ▸), *APEX2* (Bruker, 2005 ▸), *SAINT* (Bruker, 2002 ▸), *SHELXT* (Sheldrick, 2015*a* ▸), *SHELXL2014* (Sheldrick, 2015*b* ▸) and *SHELXTL* (Sheldrick, 2008 ▸).

## supplementary crystallographic information

### (1) 4-Methyl-2-oxo-2H-chromene-7,8-diyl diacetate Crystal data

**Table:**

C_14_H_12_O_6_	*Z* = 2
*M**_r_* = 276.24	*F*(000) = 288
Triclinic, *P*1	*D*_x_ = 1.401 Mg m^−^^3^
*a* = 7.3722 (10) Å	Mo *K*α radiation, λ = 0.71073 Å
*b* = 8.7235 (7) Å	Cell parameters from 1905 reflections
*c* = 11.7032 (15) Å	θ = 4.4–32.8°
α = 69.263 (10)°	µ = 0.11 mm^−^^1^
β = 87.519 (11)°	*T* = 173 K
γ = 69.113 (10)°	The symmetry employed for this shelxl refinement is uniquely defined by the following loop, which should always be used as a source of symmetry information in preference to the above space-group names. They are only intended as comments., colorless
*V* = 654.66 (14) Å^3^	0.33 × 0.26 × 0.11 mm

### (1) 4-Methyl-2-oxo-2H-chromene-7,8-diyl diacetate Data collection

**Table:**

Agilent Xcalibur Eos Gemini diffractometer	4296 independent reflections
Radiation source: Enhance (Mo) X-ray Source	3087 reflections with *I* > 2σ(*I*)
Detector resolution: 16.0416 pixels mm^-1^	*R*_int_ = 0.036
ω scans	θ_max_ = 32.7°, θ_min_ = 3.2°
Absorption correction: multi-scan (*CrysAlis PRO*; Agilent, 2014)	*h* = −11→8
*T*_min_ = 0.883, *T*_max_ = 1.000	*k* = −13→12
7360 measured reflections	*l* = −16→17

### (1) 4-Methyl-2-oxo-2H-chromene-7,8-diyl diacetate Refinement

**Table:**

Refinement on *F*^2^	13 restraints
Least-squares matrix: full	Hydrogen site location: inferred from neighbouring sites
*R*[*F*^2^ > 2σ(*F*^2^)] = 0.055	H-atom parameters constrained
*wR*(*F*^2^) = 0.156	*w* = 1/[σ^2^(*F*_o_^2^) + (0.0738*P*)^2^ + 0.0282*P*] where *P* = (*F*_o_^2^ + 2*F*_c_^2^)/3
*S* = 1.04	(Δ/σ)_max_ < 0.001
4296 reflections	Δρ_max_ = 0.36 e Å^−^^3^
192 parameters	Δρ_min_ = −0.24 e Å^−^^3^

### (1) 4-Methyl-2-oxo-2H-chromene-7,8-diyl diacetate Special details

**Table:**

Experimental. Absorption correction: CrysAlisPro (Agilent Technologies, 2014) Empirical absorption correction using spherical harmonics, implemented in SCALE3 ABSPACK scaling algorithm.
Geometry. All esds (except the esd in the dihedral angle between two l.s. planes) are estimated using the full covariance matrix. The cell esds are taken into account individually in the estimation of esds in distances, angles and torsion angles; correlations between esds in cell parameters are only used when they are defined by crystal symmetry. An approximate (isotropic) treatment of cell esds is used for estimating esds involving l.s. planes.

### (1) 4-Methyl-2-oxo-2H-chromene-7,8-diyl diacetate Fractional atomic coordinates and isotropic or equivalent isotropic displacement parameters (Å^2^)

**Table:**

	*x*	*y*	*z*	*U*_iso_*/*U*_eq_	Occ. (<1)
O1	0.48459 (13)	0.24436 (11)	0.69118 (8)	0.0225 (2)	
O2	0.67011 (16)	0.00141 (12)	0.66239 (10)	0.0336 (3)	
O3	0.09094 (15)	0.73883 (12)	0.78120 (9)	0.0264 (2)	
O4	0.20400 (17)	0.95648 (13)	0.68569 (10)	0.0332 (3)	
O5	0.38600 (14)	0.41942 (12)	0.84842 (8)	0.0238 (2)	
C2	0.54690 (19)	0.14688 (16)	0.61566 (13)	0.0234 (3)	
C3	0.4606 (2)	0.22921 (16)	0.49018 (12)	0.0235 (3)	
H3A	0.5012	0.1646	0.4372	0.028*	
C4	0.32496 (19)	0.39359 (16)	0.44454 (12)	0.0203 (2)	
C11	0.2429 (2)	0.47678 (18)	0.31289 (12)	0.0267 (3)	
H11A	0.2925	0.3903	0.2729	0.040*	
H11B	0.2823	0.5775	0.2711	0.040*	
H11C	0.1003	0.5170	0.3085	0.040*	
C10	0.26109 (18)	0.49190 (15)	0.52589 (11)	0.0183 (2)	
C5	0.11893 (18)	0.66244 (15)	0.49057 (12)	0.0209 (3)	
H5A	0.0596	0.7197	0.4086	0.025*	
C6	0.06342 (19)	0.74874 (15)	0.57236 (12)	0.0225 (3)	
H6A	−0.0339	0.8636	0.5473	0.027*	
C7	0.15204 (19)	0.66502 (15)	0.69197 (12)	0.0207 (2)	
C8	0.29286 (18)	0.49730 (15)	0.72996 (11)	0.0193 (2)	
C9	0.34563 (17)	0.41057 (14)	0.64741 (12)	0.0183 (2)	
C12	0.1158 (2)	0.89364 (16)	0.76502 (13)	0.0244 (3)	
C13	0.0176 (3)	0.9662 (2)	0.85859 (16)	0.0368 (4)	
H13A	0.0693	1.0528	0.8641	0.055*	
H13B	0.0421	0.8704	0.9386	0.055*	
H13C	−0.1231	1.0232	0.8347	0.055*	
C14	0.3169 (3)	0.3042 (2)	0.93265 (14)	0.0376 (4)	
O6A	0.1945 (8)	0.2587 (9)	0.9046 (3)	0.0509 (11)	0.755 (17)
C15A	0.4350 (12)	0.2219 (9)	1.0560 (7)	0.0572 (13)	0.755 (17)
H15A	0.5502	0.1208	1.0568	0.086*	0.755 (17)
H15B	0.3550	0.1827	1.1207	0.086*	0.755 (17)
H15C	0.4764	0.3090	1.0706	0.086*	0.755 (17)
O6B	0.150 (2)	0.3148 (19)	0.9106 (11)	0.0509 (11)	0.245 (17)
C15B	0.406 (4)	0.265 (3)	1.051 (2)	0.0572 (13)	0.245 (17)
H15D	0.3343	0.3584	1.0818	0.086*	0.245 (17)
H15E	0.5415	0.2586	1.0435	0.086*	0.245 (17)
H15F	0.4039	0.1523	1.1073	0.086*	0.245 (17)

### (1) 4-Methyl-2-oxo-2H-chromene-7,8-diyl diacetate Atomic displacement parameters (Å^2^)

**Table:**

	*U*^11^	*U*^22^	*U*^33^	*U*^12^	*U*^13^	*U*^23^
O1	0.0247 (5)	0.0181 (4)	0.0211 (5)	−0.0030 (3)	−0.0014 (4)	−0.0075 (3)
O2	0.0349 (6)	0.0229 (5)	0.0345 (6)	−0.0005 (4)	0.0013 (5)	−0.0107 (4)
O3	0.0366 (5)	0.0242 (4)	0.0236 (5)	−0.0127 (4)	0.0108 (4)	−0.0137 (4)
O4	0.0434 (6)	0.0334 (5)	0.0314 (6)	−0.0202 (5)	0.0118 (5)	−0.0162 (4)
O5	0.0286 (5)	0.0265 (4)	0.0171 (4)	−0.0115 (4)	−0.0004 (4)	−0.0069 (3)
C2	0.0247 (6)	0.0201 (5)	0.0263 (7)	−0.0074 (5)	0.0053 (5)	−0.0105 (5)
C3	0.0276 (6)	0.0242 (6)	0.0239 (7)	−0.0109 (5)	0.0064 (5)	−0.0135 (5)
C4	0.0227 (6)	0.0237 (5)	0.0196 (6)	−0.0125 (5)	0.0048 (5)	−0.0099 (5)
C11	0.0313 (7)	0.0320 (7)	0.0204 (7)	−0.0131 (6)	0.0023 (5)	−0.0118 (5)
C10	0.0194 (6)	0.0198 (5)	0.0178 (6)	−0.0091 (4)	0.0025 (4)	−0.0072 (4)
C5	0.0218 (6)	0.0207 (5)	0.0189 (6)	−0.0080 (4)	0.0007 (5)	−0.0052 (4)
C6	0.0232 (6)	0.0183 (5)	0.0236 (7)	−0.0056 (4)	0.0031 (5)	−0.0070 (5)
C7	0.0242 (6)	0.0207 (5)	0.0211 (6)	−0.0100 (5)	0.0070 (5)	−0.0109 (5)
C8	0.0217 (6)	0.0206 (5)	0.0163 (6)	−0.0092 (4)	0.0009 (4)	−0.0058 (4)
C9	0.0184 (5)	0.0157 (5)	0.0207 (6)	−0.0060 (4)	0.0016 (4)	−0.0066 (4)
C12	0.0278 (6)	0.0237 (6)	0.0240 (7)	−0.0082 (5)	0.0013 (5)	−0.0122 (5)
C13	0.0480 (9)	0.0391 (8)	0.0367 (9)	−0.0195 (7)	0.0147 (7)	−0.0266 (7)
C14	0.0538 (10)	0.0411 (8)	0.0212 (7)	−0.0277 (7)	0.0018 (7)	−0.0045 (6)
O6A	0.076 (2)	0.061 (2)	0.0308 (8)	−0.053 (2)	0.0009 (11)	−0.0047 (13)
C15A	0.088 (3)	0.055 (3)	0.0236 (12)	−0.038 (3)	−0.0136 (16)	0.006 (2)
O6B	0.076 (2)	0.061 (2)	0.0308 (8)	−0.053 (2)	0.0009 (11)	−0.0047 (13)
C15B	0.088 (3)	0.055 (3)	0.0236 (12)	−0.038 (3)	−0.0136 (16)	0.006 (2)

### (1) 4-Methyl-2-oxo-2H-chromene-7,8-diyl diacetate Geometric parameters (Å, º)

**Table:**

O1—C9	1.3691 (14)	C5—H5A	0.9500
O1—C2	1.3906 (15)	C6—C7	1.3910 (19)
O2—C2	1.2100 (16)	C6—H6A	0.9500
O3—C12	1.3731 (15)	C7—C8	1.3829 (17)
O3—C7	1.3916 (15)	C8—C9	1.3901 (17)
O4—C12	1.1945 (17)	C12—C13	1.4898 (19)
O5—C14	1.3641 (17)	C13—H13A	0.9800
O5—C8	1.3921 (15)	C13—H13B	0.9800
C2—C3	1.4440 (19)	C13—H13C	0.9800
C3—C4	1.3502 (18)	C14—O6A	1.205 (4)
C3—H3A	0.9500	C14—O6B	1.234 (14)
C4—C10	1.4544 (17)	C14—C15B	1.43 (2)
C4—C11	1.4973 (19)	C14—C15A	1.512 (7)
C11—H11A	0.9800	C15A—H15A	0.9800
C11—H11B	0.9800	C15A—H15B	0.9800
C11—H11C	0.9800	C15A—H15C	0.9800
C10—C9	1.4005 (18)	C15B—H15D	0.9800
C10—C5	1.4045 (16)	C15B—H15E	0.9800
C5—C6	1.3810 (17)	C15B—H15F	0.9800
			
C9—O1—C2	120.75 (10)	C9—C8—O5	120.50 (11)
C12—O3—C7	117.51 (10)	O1—C9—C8	116.45 (11)
C14—O5—C8	116.43 (11)	O1—C9—C10	122.58 (11)
O2—C2—O1	116.03 (12)	C8—C9—C10	120.96 (11)
O2—C2—C3	126.76 (13)	O4—C12—O3	122.90 (12)
O1—C2—C3	117.20 (11)	O4—C12—C13	126.98 (13)
C4—C3—C2	123.15 (12)	O3—C12—C13	110.12 (12)
C4—C3—H3A	118.4	C12—C13—H13A	109.5
C2—C3—H3A	118.4	C12—C13—H13B	109.5
C3—C4—C10	118.48 (12)	H13A—C13—H13B	109.5
C3—C4—C11	121.68 (12)	C12—C13—H13C	109.5
C10—C4—C11	119.83 (11)	H13A—C13—H13C	109.5
C4—C11—H11A	109.5	H13B—C13—H13C	109.5
C4—C11—H11B	109.5	O6A—C14—O5	122.2 (2)
H11A—C11—H11B	109.5	O6B—C14—O5	117.7 (6)
C4—C11—H11C	109.5	O6B—C14—C15B	125.8 (15)
H11A—C11—H11C	109.5	O5—C14—C15B	107.3 (11)
H11B—C11—H11C	109.5	O6A—C14—C15A	125.4 (4)
C9—C10—C5	118.01 (11)	O5—C14—C15A	111.6 (3)
C9—C10—C4	117.84 (11)	C14—C15A—H15A	109.5
C5—C10—C4	124.15 (12)	C14—C15A—H15B	109.5
C6—C5—C10	121.47 (12)	H15A—C15A—H15B	109.5
C6—C5—H5A	119.3	C14—C15A—H15C	109.5
C10—C5—H5A	119.3	H15A—C15A—H15C	109.5
C5—C6—C7	119.04 (11)	H15B—C15A—H15C	109.5
C5—C6—H6A	120.5	C14—C15B—H15D	109.5
C7—C6—H6A	120.5	C14—C15B—H15E	109.5
C8—C7—C6	121.09 (11)	H15D—C15B—H15E	109.5
C8—C7—O3	117.00 (11)	C14—C15B—H15F	109.5
C6—C7—O3	121.65 (11)	H15D—C15B—H15F	109.5
C7—C8—C9	119.41 (11)	H15E—C15B—H15F	109.5
C7—C8—O5	120.05 (11)		
			
C9—O1—C2—O2	180.00 (11)	O3—C7—C8—O5	−8.72 (17)
C9—O1—C2—C3	0.67 (17)	C14—O5—C8—C7	98.60 (15)
O2—C2—C3—C4	−179.19 (13)	C14—O5—C8—C9	−83.97 (15)
O1—C2—C3—C4	0.07 (19)	C2—O1—C9—C8	179.93 (11)
C2—C3—C4—C10	−0.66 (19)	C2—O1—C9—C10	−0.79 (17)
C2—C3—C4—C11	178.16 (12)	C7—C8—C9—O1	−179.44 (10)
C3—C4—C10—C9	0.54 (17)	O5—C8—C9—O1	3.11 (17)
C11—C4—C10—C9	−178.31 (11)	C7—C8—C9—C10	1.28 (18)
C3—C4—C10—C5	−178.89 (11)	O5—C8—C9—C10	−176.18 (10)
C11—C4—C10—C5	2.27 (19)	C5—C10—C9—O1	179.64 (10)
C9—C10—C5—C6	0.16 (18)	C4—C10—C9—O1	0.18 (18)
C4—C10—C5—C6	179.58 (11)	C5—C10—C9—C8	−1.12 (18)
C10—C5—C6—C7	0.63 (18)	C4—C10—C9—C8	179.42 (11)
C5—C6—C7—C8	−0.48 (19)	C7—O3—C12—O4	−7.8 (2)
C5—C6—C7—O3	−174.49 (11)	C7—O3—C12—C13	171.97 (12)
C12—O3—C7—C8	120.51 (13)	C8—O5—C14—O6A	6.8 (5)
C12—O3—C7—C6	−65.25 (16)	C8—O5—C14—O6B	−20.6 (8)
C6—C7—C8—C9	−0.46 (18)	C8—O5—C14—C15B	−169.5 (13)
O3—C7—C8—C9	173.82 (11)	C8—O5—C14—C15A	177.4 (4)
C6—C7—C8—O5	177.00 (11)		

### (1) 4-Methyl-2-oxo-2H-chromene-7,8-diyl diacetate Hydrogen-bond geometry (Å, º)

**Table:**

*D*—H···*A*	*D*—H	H···*A*	*D*···*A*	*D*—H···*A*
C6—H6*A*···O2^i^	0.95	2.65	3.3465 (17)	130
C13—H13*A*···O6*A*^ii^	0.98	2.48	3.451 (5)	173
C15*A*—H15*B*···O2^iii^	0.98	2.52	3.401 (8)	150

Symmetry codes: (i) *x*−1, *y*+1, *z*; (ii) *x*, *y*+1, *z*; (iii) −*x*+1, −*y*, −*z*+2.

### (2) 4-Methyl-2-oxo-2H-chromene-7,8-diyl bis(pent-4-ynoate) Crystal data

**Table:**

C_20_H_16_O_6_	*F*(000) = 736
*M**_r_* = 352.33	*D*_x_ = 1.357 Mg m^−^^3^
Monoclinic, *P*2_1_/*n*	Mo *K*α radiation, λ = 0.71073 Å
*a* = 5.2785 (3) Å	Cell parameters from 9562 reflections
*b* = 16.3785 (8) Å	θ = 2.5–30.4°
*c* = 20.0502 (11) Å	µ = 0.10 mm^−^^1^
β = 95.992 (2)°	*T* = 200 K
*V* = 1723.95 (16) Å^3^	Rod, colourless
*Z* = 4	0.55 × 0.14 × 0.11 mm

### (2) 4-Methyl-2-oxo-2H-chromene-7,8-diyl bis(pent-4-ynoate) Data collection

**Table:**

Bruker Quest diffractometer	3859 reflections with *I* > 2σ(*I*)
ω scans	*R*_int_ = 0.035
Absorption correction: multi-scan (*SADABS*; Sheldrick, 1996)	θ_max_ = 30.6°, θ_min_ = 2.5°
*T*_min_ = 0.658, *T*_max_ = 0.746	*h* = −7→6
24358 measured reflections	*k* = −23→23
5276 independent reflections	*l* = −28→28

### (2) 4-Methyl-2-oxo-2H-chromene-7,8-diyl bis(pent-4-ynoate) Refinement

**Table:**

Refinement on *F*^2^	13 restraints
Least-squares matrix: full	Hydrogen site location: mixed
*R*[*F*^2^ > 2σ(*F*^2^)] = 0.057	H-atom parameters constrained
*wR*(*F*^2^) = 0.142	*w* = 1/[σ^2^(*F*_o_^2^) + (0.0483*P*)^2^ + 1.0298*P*] where *P* = (*F*_o_^2^ + 2*F*_c_^2^)/3
*S* = 1.07	(Δ/σ)_max_ < 0.001
5276 reflections	Δρ_max_ = 0.37 e Å^−^^3^
255 parameters	Δρ_min_ = −0.21 e Å^−^^3^

### (2) 4-Methyl-2-oxo-2H-chromene-7,8-diyl bis(pent-4-ynoate) Special details

**Table:**

Geometry. All esds (except the esd in the dihedral angle between two l.s. planes) are estimated using the full covariance matrix. The cell esds are taken into account individually in the estimation of esds in distances, angles and torsion angles; correlations between esds in cell parameters are only used when they are defined by crystal symmetry. An approximate (isotropic) treatment of cell esds is used for estimating esds involving l.s. planes.

### (2) 4-Methyl-2-oxo-2H-chromene-7,8-diyl bis(pent-4-ynoate) Fractional atomic coordinates and isotropic or equivalent isotropic displacement parameters (Å^2^)

**Table:**

	*x*	*y*	*z*	*U*_iso_*/*U*_eq_	Occ. (<1)
O1	0.4888 (2)	0.14589 (6)	0.70580 (6)	0.0315 (3)	
O2	0.7381 (3)	0.03765 (8)	0.70909 (7)	0.0482 (4)	
O3	−0.0447 (2)	0.37392 (7)	0.67567 (6)	0.0326 (3)	
O4	0.1091 (2)	0.50086 (8)	0.66067 (7)	0.0416 (3)	
O5	0.0579 (2)	0.21864 (7)	0.64977 (5)	0.0278 (2)	
O6	0.3186 (2)	0.22167 (8)	0.56756 (6)	0.0384 (3)	
C2	0.7046 (3)	0.10414 (10)	0.73219 (8)	0.0329 (3)	
C3	0.8638 (3)	0.14369 (10)	0.78577 (8)	0.0327 (3)	
H3A	1.0078	0.1150	0.8063	0.039*	
C4	0.8176 (3)	0.21931 (10)	0.80793 (7)	0.0289 (3)	
C5	0.5425 (3)	0.34519 (9)	0.79035 (8)	0.0309 (3)	
H5A	0.6481	0.3735	0.8240	0.037*	
C6	0.3343 (3)	0.38464 (10)	0.75779 (8)	0.0317 (3)	
H6A	0.2971	0.4395	0.7686	0.038*	
C7	0.1793 (3)	0.34264 (9)	0.70865 (7)	0.0272 (3)	
C8	0.2314 (3)	0.26251 (9)	0.69265 (7)	0.0249 (3)	
C9	0.4453 (3)	0.22431 (9)	0.72504 (7)	0.0253 (3)	
C10	0.6024 (3)	0.26439 (9)	0.77514 (7)	0.0263 (3)	
C11	0.9821 (4)	0.25697 (12)	0.86536 (9)	0.0394 (4)	
H11D	1.1244	0.2203	0.8794	0.056 (6)*	
H11E	0.8808	0.2659	0.9030	0.066 (7)*	
H11F	1.0484	0.3094	0.8512	0.068 (7)*	
C12	−0.0603 (3)	0.45274 (10)	0.65301 (8)	0.0300 (3)	
C13	−0.3222 (3)	0.46849 (11)	0.61876 (10)	0.0394 (4)	
H13A	−0.3207	0.5209	0.5942	0.047*	
H13B	−0.4420	0.4744	0.6533	0.047*	
C14	−0.4205 (4)	0.40154 (13)	0.56970 (11)	0.0449 (5)	
H14A	−0.591 (5)	0.4172 (15)	0.5499 (13)	0.067 (7)*	
H14B	−0.433 (4)	0.3504 (13)	0.5928 (11)	0.042 (5)*	
C15A	−0.280 (2)	0.3949 (8)	0.5166 (7)	0.0399 (16)	0.55 (2)
C16A	−0.147 (3)	0.3912 (8)	0.4725 (6)	0.059 (2)	0.55 (2)
H16A	−0.0410	0.3882	0.4371	0.071*	0.55 (2)
C15B	−0.228 (3)	0.3822 (10)	0.5170 (8)	0.0399 (16)	0.45 (2)
C16B	−0.084 (3)	0.3704 (10)	0.4775 (8)	0.059 (2)	0.45 (2)
H16B	0.0337	0.3609	0.4455	0.071*	0.45 (2)
C17	0.1254 (3)	0.19905 (9)	0.58756 (7)	0.0258 (3)	
C18	−0.0746 (3)	0.14582 (10)	0.55133 (8)	0.0300 (3)	
H18A	−0.2420	0.1734	0.5498	0.036*	
H18B	−0.0856	0.0939	0.5762	0.036*	
C19	−0.0149 (3)	0.12754 (10)	0.48024 (8)	0.0341 (4)	
H19A	−0.0120	0.1794	0.4549	0.041*	
H19B	0.1567	0.1028	0.4819	0.041*	
C20	−0.2013 (4)	0.07199 (10)	0.44457 (8)	0.0360 (4)	
C21	−0.3513 (4)	0.02838 (12)	0.41527 (10)	0.0468 (5)	
H21	−0.4724	−0.0068	0.3916	0.077 (8)*	

### (2) 4-Methyl-2-oxo-2H-chromene-7,8-diyl bis(pent-4-ynoate) Atomic displacement parameters (Å^2^)

**Table:**

	*U*^11^	*U*^22^	*U*^33^	*U*^12^	*U*^13^	*U*^23^
O1	0.0352 (6)	0.0212 (5)	0.0355 (6)	−0.0018 (4)	−0.0082 (5)	−0.0036 (4)
O2	0.0545 (8)	0.0302 (6)	0.0553 (8)	0.0084 (6)	−0.0157 (6)	−0.0085 (6)
O3	0.0307 (6)	0.0262 (6)	0.0396 (6)	−0.0003 (4)	−0.0019 (5)	−0.0005 (5)
O4	0.0330 (6)	0.0369 (7)	0.0538 (8)	−0.0062 (5)	−0.0014 (5)	0.0089 (6)
O5	0.0264 (5)	0.0289 (5)	0.0273 (5)	−0.0056 (4)	−0.0008 (4)	−0.0043 (4)
O6	0.0324 (6)	0.0449 (7)	0.0383 (6)	−0.0130 (5)	0.0061 (5)	−0.0084 (5)
C2	0.0373 (9)	0.0238 (7)	0.0358 (8)	−0.0007 (6)	−0.0042 (7)	0.0024 (6)
C3	0.0333 (8)	0.0288 (8)	0.0341 (8)	−0.0035 (6)	−0.0057 (6)	0.0062 (6)
C4	0.0309 (8)	0.0293 (7)	0.0253 (7)	−0.0089 (6)	−0.0025 (6)	0.0049 (6)
C5	0.0413 (9)	0.0268 (7)	0.0233 (7)	−0.0087 (6)	−0.0024 (6)	−0.0037 (6)
C6	0.0427 (9)	0.0246 (7)	0.0272 (7)	−0.0031 (6)	0.0009 (6)	−0.0033 (6)
C7	0.0307 (7)	0.0258 (7)	0.0250 (7)	−0.0014 (6)	0.0024 (6)	0.0006 (5)
C8	0.0276 (7)	0.0239 (7)	0.0227 (6)	−0.0071 (6)	0.0001 (5)	−0.0018 (5)
C9	0.0312 (7)	0.0192 (6)	0.0249 (7)	−0.0052 (6)	0.0001 (5)	0.0002 (5)
C10	0.0315 (7)	0.0239 (7)	0.0224 (7)	−0.0072 (6)	−0.0018 (5)	0.0015 (5)
C11	0.0410 (9)	0.0414 (10)	0.0324 (8)	−0.0080 (8)	−0.0120 (7)	0.0010 (7)
C12	0.0300 (8)	0.0283 (8)	0.0323 (8)	0.0030 (6)	0.0063 (6)	−0.0008 (6)
C13	0.0309 (8)	0.0318 (9)	0.0544 (11)	0.0021 (7)	−0.0004 (7)	−0.0005 (7)
C14	0.0344 (9)	0.0408 (10)	0.0570 (12)	−0.0078 (8)	−0.0078 (8)	−0.0006 (9)
C15A	0.043 (4)	0.034 (4)	0.0394 (10)	−0.006 (3)	−0.008 (2)	−0.0004 (19)
C16A	0.071 (5)	0.057 (5)	0.051 (2)	−0.017 (3)	0.008 (3)	−0.008 (3)
C15B	0.043 (4)	0.034 (4)	0.0394 (10)	−0.006 (3)	−0.008 (2)	−0.0004 (19)
C16B	0.071 (5)	0.057 (5)	0.051 (2)	−0.017 (3)	0.008 (3)	−0.008 (3)
C17	0.0260 (7)	0.0229 (7)	0.0274 (7)	0.0005 (5)	−0.0014 (5)	−0.0015 (5)
C18	0.0280 (7)	0.0308 (8)	0.0305 (8)	−0.0053 (6)	−0.0002 (6)	−0.0060 (6)
C19	0.0399 (9)	0.0331 (8)	0.0282 (8)	−0.0055 (7)	−0.0009 (6)	−0.0013 (6)
C20	0.0480 (10)	0.0307 (8)	0.0275 (8)	0.0009 (7)	−0.0041 (7)	0.0001 (6)
C21	0.0602 (12)	0.0379 (10)	0.0390 (10)	−0.0072 (9)	−0.0103 (9)	−0.0037 (8)

### (2) 4-Methyl-2-oxo-2H-chromene-7,8-diyl bis(pent-4-ynoate) Geometric parameters (Å, º)

**Table:**

O1—C9	1.3675 (18)	C11—H11E	0.9800
O1—C2	1.3851 (19)	C11—H11F	0.9800
O2—C2	1.204 (2)	C12—C13	1.501 (2)
O3—C12	1.3682 (19)	C13—C14	1.527 (3)
O3—C7	1.3911 (19)	C13—H13A	0.9900
O4—C12	1.189 (2)	C13—H13B	0.9900
O5—C17	1.3705 (18)	C14—C15A	1.364 (13)
O5—C8	1.3889 (17)	C14—C15B	1.574 (15)
O6—C17	1.1931 (19)	C14—H14A	0.98 (3)
C2—C3	1.446 (2)	C14—H14B	0.96 (2)
C3—C4	1.347 (2)	C15A—C16A	1.186 (9)
C3—H3A	0.9500	C16A—H16A	0.9500
C4—C10	1.453 (2)	C15B—C16B	1.169 (11)
C4—C11	1.501 (2)	C16B—H16B	0.9500
C5—C6	1.379 (2)	C17—C18	1.497 (2)
C5—C10	1.402 (2)	C18—C19	1.521 (2)
C5—H5A	0.9500	C18—H18A	0.9900
C6—C7	1.394 (2)	C18—H18B	0.9900
C6—H6A	0.9500	C19—C20	1.470 (2)
C7—C8	1.385 (2)	C19—H19A	0.9900
C8—C9	1.391 (2)	C19—H19B	0.9900
C9—C10	1.3977 (19)	C20—C21	1.177 (3)
C11—H11D	0.9800	C21—H21	0.9500
			
C9—O1—C2	120.77 (12)	O3—C12—C13	109.55 (14)
C12—O3—C7	121.60 (12)	C12—C13—C14	113.95 (15)
C17—O5—C8	117.86 (11)	C12—C13—H13A	108.8
O2—C2—O1	116.59 (14)	C14—C13—H13A	108.8
O2—C2—C3	126.42 (16)	C12—C13—H13B	108.8
O1—C2—C3	116.97 (14)	C14—C13—H13B	108.8
C4—C3—C2	123.07 (15)	H13A—C13—H13B	107.7
C4—C3—H3A	118.5	C15A—C14—C13	112.6 (6)
C2—C3—H3A	118.5	C13—C14—C15B	112.2 (7)
C3—C4—C10	118.56 (14)	C15A—C14—H14A	104.9 (16)
C3—C4—C11	121.39 (15)	C13—C14—H14A	107.9 (15)
C10—C4—C11	120.05 (14)	C15B—C14—H14A	114.3 (16)
C6—C5—C10	121.75 (14)	C15A—C14—H14B	112.2 (14)
C6—C5—H5A	119.1	C13—C14—H14B	110.5 (13)
C10—C5—H5A	119.1	C15B—C14—H14B	103.3 (14)
C5—C6—C7	118.88 (14)	H14A—C14—H14B	108.4 (19)
C5—C6—H6A	120.6	C16A—C15A—C14	176.4 (14)
C7—C6—H6A	120.6	C15A—C16A—H16A	180.0
C8—C7—O3	114.71 (13)	C16B—C15B—C14	177.9 (16)
C8—C7—C6	121.01 (14)	C15B—C16B—H16B	180.0
O3—C7—C6	124.13 (14)	O6—C17—O5	123.07 (13)
C7—C8—O5	119.98 (13)	O6—C17—C18	127.00 (14)
C7—C8—C9	119.26 (13)	O5—C17—C18	109.93 (13)
O5—C8—C9	120.45 (13)	C17—C18—C19	111.39 (13)
O1—C9—C8	116.28 (12)	C17—C18—H18A	109.4
O1—C9—C10	122.63 (14)	C19—C18—H18A	109.4
C8—C9—C10	121.07 (13)	C17—C18—H18B	109.4
C9—C10—C5	117.99 (14)	C19—C18—H18B	109.4
C9—C10—C4	117.62 (14)	H18A—C18—H18B	108.0
C5—C10—C4	124.39 (13)	C20—C19—C18	112.58 (14)
C4—C11—H11D	109.5	C20—C19—H19A	109.1
C4—C11—H11E	109.5	C18—C19—H19A	109.1
H11D—C11—H11E	109.5	C20—C19—H19B	109.1
C4—C11—H11F	109.5	C18—C19—H19B	109.1
H11D—C11—H11F	109.5	H19A—C19—H19B	107.8
H11E—C11—H11F	109.5	C21—C20—C19	179.00 (19)
O4—C12—O3	124.34 (15)	C20—C21—H21	180.0
O4—C12—C13	126.10 (15)		
			
C9—O1—C2—O2	−174.76 (15)	O5—C8—C9—C10	−171.09 (13)
C9—O1—C2—C3	6.7 (2)	O1—C9—C10—C5	179.15 (14)
O2—C2—C3—C4	178.29 (18)	C8—C9—C10—C5	−2.0 (2)
O1—C2—C3—C4	−3.3 (2)	O1—C9—C10—C4	−0.8 (2)
C2—C3—C4—C10	−2.0 (2)	C8—C9—C10—C4	178.12 (13)
C2—C3—C4—C11	177.73 (16)	C6—C5—C10—C9	0.6 (2)
C10—C5—C6—C7	0.3 (2)	C6—C5—C10—C4	−179.52 (15)
C12—O3—C7—C8	−141.36 (14)	C3—C4—C10—C9	4.1 (2)
C12—O3—C7—C6	43.1 (2)	C11—C4—C10—C9	−175.69 (14)
C5—C6—C7—C8	0.3 (2)	C3—C4—C10—C5	−175.83 (15)
C5—C6—C7—O3	175.49 (14)	C11—C4—C10—C5	4.4 (2)
O3—C7—C8—O5	−3.7 (2)	C7—O3—C12—O4	−1.9 (2)
C6—C7—C8—O5	171.99 (14)	C7—O3—C12—C13	179.10 (14)
O3—C7—C8—C9	−177.27 (13)	O4—C12—C13—C14	134.73 (19)
C6—C7—C8—C9	−1.6 (2)	O3—C12—C13—C14	−46.3 (2)
C17—O5—C8—C7	111.09 (16)	C12—C13—C14—C15A	−64.6 (6)
C17—O5—C8—C9	−75.36 (17)	C12—C13—C14—C15B	−53.0 (7)
C2—O1—C9—C8	176.26 (14)	C8—O5—C17—O6	−4.2 (2)
C2—O1—C9—C10	−4.8 (2)	C8—O5—C17—C18	174.99 (12)
C7—C8—C9—O1	−178.55 (13)	O6—C17—C18—C19	−4.7 (2)
O5—C8—C9—O1	7.9 (2)	O5—C17—C18—C19	176.16 (13)
C7—C8—C9—C10	2.5 (2)	C17—C18—C19—C20	177.12 (14)

### (2) 4-Methyl-2-oxo-2H-chromene-7,8-diyl bis(pent-4-ynoate) Hydrogen-bond geometry (Å, º)

**Table:**

*D*—H···*A*	*D*—H	H···*A*	*D*···*A*	*D*—H···*A*
C13—H13*B*···O4^i^	0.99	2.43	3.244 (2)	139
C18—H18*A*···O6^i^	0.99	2.51	3.482 (2)	167

Symmetry code: (i) *x*−1, *y*, *z*.
